# Antimicrobial Therapy for Pneumonia or Fluid Overload?

**DOI:** 10.7759/cureus.12719

**Published:** 2021-01-15

**Authors:** Uche J Mbadugha, Salim Surani, Nana Akuffo, George Udeani

**Affiliations:** 1 Pharmacy, Corpus Christi Medical Center, Corpus Christi, USA; 2 Internal Medicine, Corpus Christi Medical Center, Corpus Christi, USA; 3 Internal Medicine, University of North Texas, Dallas, USA; 4 Pharmacy, Hospital Corporation of America, Houston, USA; 5 Pharmacy, College of Pharmacy, Texas A&M University, Kingsville, USA

**Keywords:** pneumonia, fluid overload, heart failure, antimicrobial therapy

## Abstract

Patients with evidence of fluid overload or heart failure (HF) without clinical symptoms of pneumonia are often treated with antimicrobial therapy for pneumonia. We conducted a retrospective study to evaluate the use of antimicrobial therapy in critically ill patients with fluid overload or heart failure diagnosed as pneumonia.

A retrospective chart review of patients on antimicrobial therapy treated for pneumonia in the intensive care unit was conducted. The study's primary outcome was the number of cases with no evidence of pneumonia, including fluid overload or heart failure, managed with antimicrobial therapy for pneumonia. Patients on antimicrobial therapy for other infections were excluded. Appropriateness of antimicrobial therapy was based on radiographic evidence, clinical data, and presentation. Patient group categories were A (pneumonia) and B (no evidence of pneumonia, fluid overload, and heart failure). Based on the subdivision of patients in Group B, where there was no evidence of pneumonia, we further classified it into two subgroups: heart failure (HF)/fluid overload (Group B1) and no evidence of HF or fluid overload (Group B2). Patients with evidence of pneumonia (Group A) were compared to the group with fluid overload and heart failure (Group B1). A p-value of < 0.05% was considered significant for detecting statistical difference.

Post-screening, data on 56 patients were collected for the study and analyzed. Mean body temperature and white blood cell count were 37.6 + 0.6^ o^C, and 17.4 + 6.88 x10^3^ µL, respectively. Based on radiographic evidence, clinical data, and presentation, 29 (52%) were classified under Group A, while 27 (48%) were classified under Group B. Median brain natriuretic peptide (BNP) for Group A vs. Group B was 514 (IQR: 1077) vs. 758 (2212) pg/mL p=0.14. The median duration of inpatient antimicrobial therapy was 7 (interquartile range [IQR]: 6) vs. 6 (IQR: 4) days, p=0.52, while the median duration of the total (inpatient and discharge prescription) antimicrobial therapy was 11 (IQR: 6) vs. 11 (IQR: 5), p=0.21. Patients with evidence of pneumonia (Group A) were compared to the group with fluid overload and heart failure (Group B1). The median BNP for the two groups was 514 (IQR: 1077) vs. 1040 (2094) pg/mL, p=0.04. Patients with documented echocardiographic evidence of ejection fraction < 55% were 4 vs. 14 for Groups A and B1, respectively. Additionally, the median BNP for Group A vs. Group B2 was 514 (IQR: 1077) vs. 189 (418) pg/mL, p=0.02.

These findings demonstrate a 48% inappropriate use of antimicrobial therapy in patients with congestive heart failure (CHF), or fluid congestion misdiagnosed as pneumonia. There was a significant difference in the median BNP observed in patients with pneumonia compared to those with fluid overload and heart failure treated as pneumonia. More cases of patients with elevated BNP and reduced left ventricular ejection fraction (LVEF) were observed in patients with fluid overload or CHF treated as pneumonia than those diagnosed with pneumonia alone. Appropriate interpretation of radiographic evidence, laboratory data, and critical clinical assessment for the use of empiric antimicrobial therapy in this population is warranted.

## Introduction

Pneumonia is the most common reason for hospital admissions in adults in the United States, apart from women giving birth. Each year, approximately one million adult patients seek hospital treatment secondary to pneumonia, with 50,000 associated deaths [1].

Pneumonia can be potentially life-threatening if appropriate guideline-directed antimicrobial therapy is not initiated promptly [2]. Classical clinical features of pneumonia include cough, fever, pleuritic chest pain, dyspnea, sputum production, and shortness of breath [3, 4]. Clinical evaluation is the first step in diagnosing pneumonia, in addition to the use of a chest X-ray.

Chest radiography (chest X-ray) is a non-invasive approach that uses small doses of ionizing radiation to project a radiographic image of the chest [5]. This creates a structural film of the internal chest anatomy, making visible the heart, lungs, bones, diaphragm, and blood vessels to further investigate heart or lung diseases. Interpretation of these radiographic images is used to evaluate and diagnose certain conditions such as pneumonia, tuberculosis, heart failure, pulmonary edema, lung cancer, and chronic lung diseases. Clinicians employ the use of chest radiographic images correlated with physical examinations and clinical findings to make appropriate diagnoses. The knowledge acquired through this process aids clinicians in identifying and initiate appropriate guideline-recommended treatment options. 

The Infectious Diseases Society of America (IDSA) recommends the use of clinical presentation. A demonstrable infiltrate by chest radiograph or other imaging technique, with or without supporting microbiological data, for the diagnosis of pneumonia, is also recommended [3, 4, 6]. Chest radiography is the preferred method for initial imaging, while the CT scan or magnetic imaging is reserved for further structural evaluation, which includes detecting cavitation, or adenopathy. The radiographic appearance of community-acquired pneumonia may include lobar consolidation, interstitial infiltrates, and cavitation [4].

Initiation of empiric antimicrobial therapy and supportive therapy is recommended to treat pneumonia, based on patients' presentation and risk factors. Current literature recommends seven days of therapy once the etiology of pneumonia has been established via reliable microbiology data. This should depend upon the rate of improvement of clinical, radiologic, and laboratory parameters and the type of pneumonia [3, 6]. Switch from intravenous to oral therapy and de-escalation when patients become both clinically and hemodynamically stable are essential components of good antimicrobial stewardship in pneumonia patients [3]. Antimicrobial stewardship programs (ASP) provide an effective means for communicating de-escalation of antimicrobial therapy. This has been proven to decrease antimicrobial resistance, costs, and adverse effects [7, 8]. 

Heart failure (HF) is a significant health problem, with a prevalence of about 6.2 million and estimated costs of $30 million annually in the United States [9, 10]. The incidence of heart failure increases with age, and approximately half of individuals who develop heart failure die within five years of diagnosis [11, 12]. Initial evaluation of acute decompensated heart failure includes a thorough history and physical examination. This includes taking into consideration volume status, vital signs, clinical laboratory data, and biomarkers, such as brain natriuretic peptide (BNP) or N-terminal pro-B-type natriuretic peptide (NT-proBNP) [13]. In addition to these parameters, the American Heart Association recommends that chest X-ray be utilized in patients with suspected or new-onset HF or acute decompensated HF. This is essential to assess heart size and pulmonary congestion, detect other cardiac, pulmonary, and associated diseases that may cause or contribute to symptoms [13]. A two-dimensional echocardiogram is used to assess ventricular function, size, wall thickness/motion, and valve function. Guideline-directed medical therapy should be employed to improve symptoms and reduce the progression of heart failure [13].

A Henry Ford Health System study demonstrated that 72% of patients were misdiagnosed with pneumonia upon readmission into their institution. Notably, 72% of the misdiagnoses occurred in the emergency department (ED). According to the study, misdiagnoses of pneumonia led to overuse of antimicrobial agents, resulting in increased healthcare costs [14]. Current guidelines for community-acquired pneumonia recommend repeat imaging (chest X-ray) within 24-48 hours post-initiation of empiric antibiotics in patients hospitalized for suspected pneumonia with initial negative chest radiographic findings [3]. Subsequent chest X-rays can be used to differentiate between the diagnosis of suspected pneumonia and fluid overload and the selection of appropriate management for the patient. Empiric antimicrobial therapy can be discontinued if no pneumonia symptoms are observed or negative radiographic evidence exists from repeat X-rays.

Antibiotic stewardship is designed to encourage the appropriate use of antimicrobial agents by promoting optimal drug regimen selection, including dosing, duration of therapy, and administration route [7, 8]. Our institution has established an antimicrobial stewardship program that is championed by an infectious disease physician and co-led by a clinical pharmacist. ASP rounds are conducted bi-weekly at the intensive care units (ICUs) to assess the appropriate use of antimicrobial therapy and make pertinent recommendations to improve patient outcomes. Management of pneumonia differs from that of non-infectious cardiac or pulmonary disease such as fluid overload and emphysema. Therefore, it is imperative to appropriately interpret chest radiographic images to treat pneumonia to improve treatment outcomes effectively. Patients with radiographic evidence of fluid overload without typical pneumonia symptoms are often misdiagnosed and initiated on empiric antimicrobial therapy. Regular monitoring of clinical presentation, subsequent chest X-rays, and cultures can guide in de-escalation or change in antimicrobial therapy. Inappropriate use of antimicrobial therapy in such clinical settings can lead to an increased risk of *Clostridium difficile* infection, antimicrobial resistance, and prolonged hospitalization [7]. On the other hand, the inadequate management of heart failure contributes to increased hospitalization rates and the financial burden to all parties, including patient, hospital, and third-party payers, and mortality [13]. It is critical that these disease states are appropriately diagnosed and appropriate interventions rendered to this category of patients.

This study's primary goal is to evaluate the use of antimicrobial therapy in critically ill patients with fluid overload or heart failure diagnosed as pneumonia.

## Materials and methods

Study design and population

A retrospective study of patients in the intensive care unit (ICU) with abnormal chest X-ray findings treated with antimicrobial therapy for pneumonia was conducted at our institution. The study protocol was reviewed and approved by the Institutional Review Board. Data of patients admitted into the ICU with abnormal chest X-ray findings on antimicrobial therapy from June through September 2016 was generated through the electronic medical records (EMR). Patients were eligible if they were 18 or older, not pregnant, and not on antimicrobial therapy for other infections other than pneumonia. Antibiotic use for other concurrent infections was excluded because they could confound evaluations of patients treated for pneumonia.

Data collection

All patients admitted to the ICU and treated with antimicrobial therapy for pneumonia during the study period were identified and evaluated. Data collected and analyzed included demographics, clinical presentation, vital signs, laboratory - microbiology, and radiographic information. The study was conducted at a community hospital in Corpus Christi, Texas, United States. Here, critically ill patients are routinely admitted into the ICU, with empiric antibiotics initiated in cases of suspected infections. Upon routine further evaluation by clinicians and AST, antimicrobial therapy is either continued, de-escalated, or discontinued.

**Figure 1:**
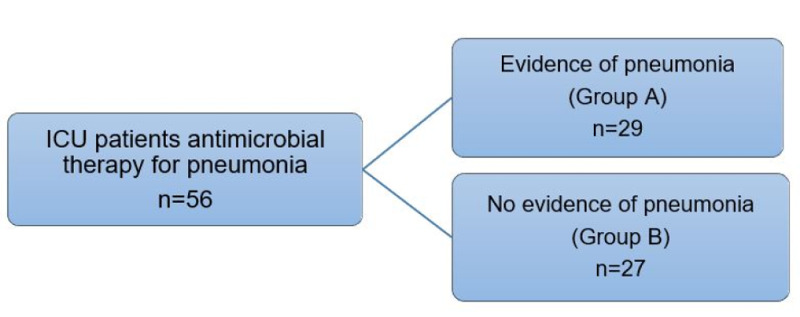
Control group (Group A) is defined as patients with radiographic evidence and clinical symptoms of pneumonia diagnosed with pneumonia. Case group (Group B) is defined as patients with no evidence of pneumonia, which includes evidence of fluid congestion and heart failure diagnosed with pneumonia ICU: intensive care unit

In our study population, antimicrobial therapy was evaluated for appropriateness based on radiographic evidence and clinical presentation following current treatment practice guidelines for pneumonia. Patients were assigned to one of two groups, as illustrated in Figure 1. The control group (Group A) was defined as patients with radiographic and clinical symptoms of pneumonia. The case group (Group B) consisted of patients with no evidence of pneumonia, had evidence of either fluid congestion or heart failure, misdiagnosed with pneumonia. Initial chest X-rays were obtained upon admission and as deemed clinically necessary throughout the hospitalization. 

Outcome measures

The study's primary outcome was the number of patients without evidence of pneumonia, including fluid overload or heart failure managed with antimicrobial therapy for pneumonia. The secondary outcomes included duration of antimicrobial treatment, length of stay, BNP, LVEF, and the incidence of antimicrobial therapy-associated complications such as *Clostridium difficile* diarrhea.

Data analysis

Data were analyzed using descriptive statistics for the patient characteristics, frequencies for categorical data, means, and standard deviations (SDs) for continuous data. Primary variables between the two groups were compared using the Mann-Whitney U test for continuous variables and median (interquartile range: IQR). This was used to determine if any difference existed between the two study groups for the secondary outcomes. All statistical analyses were performed with GraphPad Software (San Diego, California, USA) [15]. 

## Results

Overall, 56 patients who met the study criteria and were included in the study. Of those, 56 (100%) were diagnosed with pneumonia and managed with antimicrobial therapy, and 32 (57%) were male. The median age was 69 (IQR: 17), mean body temperature and white blood cell count were 37.6 + 0.6 ^o^C, and 17.4 + 6.88 x10^3^ µL, respectively. The mean duration of inpatient antimicrobial therapy was 7.7 + 4.46 days, and the mean duration of total (inpatient and outpatient) therapy was 10.9 + 4.58 days. The median BNP and median procalcitonin levels were 578 (IQR: 1524) pg/mL and 1.25 (IQR: 5.05), respectively (Table 1). Five (8.9 %) patients had positive urine antigen tests, including Streptococcus pneumonia, Chlamydia pneumonia, and Mycoplasma pneumonia. Eleven (19%) patients had positive sputum and bronchial washings culture results (Table 2). 

**Table 1:** Demographic and clinical parameters

Patient characteristics	Eligible patients N=56
Age (years), Median (Q1-Q3)	69 (60-77)
Male, N (%) Female, N (%)	32 (57) 24 (43)
Duration of inpatient antimicrobial therapy (days), Mean + SD	7.7 + 4.46
Duration of antimicrobial therapy (inpatient and outpatient) (days), Mean + SD	10.9 + 4.58
Hospital length of stay (days), Mean + SD	9 + 7.09
Body temperature (^O^C), Mean + SD	37.6 + 0.6
White blood cell count (10^3^ µL), Mean + SD	17.4 + 6.88
Brain natriuretic peptide (pg/mL), Mean + SD Median (Q1-Q3)	578 (216-1740)
Procalcitonin (ng/mL), Median (Q1-Q3)	1.25 (0.23-5.28)
Positive sputum cultures and bronchial washings Urine antigen tests	11 (19) 5 (8.9)
Positive Clostridium difficile toxin	1 (1.8)

**Table 2:** Culture results MDRO: multidrug-resistant organism; MRSA: Methicillin-resistant *Staphylococcus aureus*

Number of patients	Sputum culture (+)	Bronchial washings (+)
1	E.Coli (MDRO)	-
1	-	Mycobacterium abscessus
2	Staphylococcus aureus	-
1	MRSA	-
1	-	Acinetobacter baumanii
2	-	Mycobacterium fortuitum
1	Pseudomonas aeruginosa	Pseudomonas aeruginosa
1	-	Klebsiella pneumonia
1	Candida albicans	-
1	Influenza B	

Twenty-nine patients (52%) were classified under Group A as those with evidence of pneumonia. In comparison, 27 (48%) were classified under Group B as patients with no evidence of pneumonia. The median BNP for Group A versus Group B was 514 (IQR: 1077) vs. 758 (2212) pg/mL, p=0.14. The median duration of inpatient antimicrobial therapy for Group A vs. Group B was 7 (IQR: 6) vs. 6 (IQR:4) days, p=0.52. In contrast, the median duration of the total (inpatient orders and discharge prescriptions) antimicrobial therapy for Group A vs. Group B was 11 (IQR: 6) vs. 11 (IQR: 5), p=0.21. The median duration of hospital length of stay for Group A vs. group B was 9 (IQR: 8.5) vs. 6 (IQR:7) days, p=0.21. (Table 3).

**Table 3:** Analysis of antimicrobial duration, length of stay, and pertinent clinical parameters in study groups BNP: brain natriuretic peptide

Data assessed	Evidence of pneumonia (Group A)	No evidence of pneumonia (Group B)	P-value
Patients, N (%)	29 (52)	27 (48)	N/A
BNP (pg/mL)	514 (141-1218)	758 (288-2500)	0.14
Duration of inpatient antimicrobial therapy (days)	7 (4-10)	6 (5-9)	0.52
Total duration of antimicrobial therapy (days)	11 (8-14)	11 (8-13)	0.63
Hospital length of stay (days)	9 (4-12.5)	6 (4-11)	0.21
Procalcitonin (ng/mL)	4.78 (0.18-12.78)	0.42 (0.16-2.58)	0.3
Positive Clostridium difficile toxin	-	1 (1.78%)	N/A

**Figure 2:**
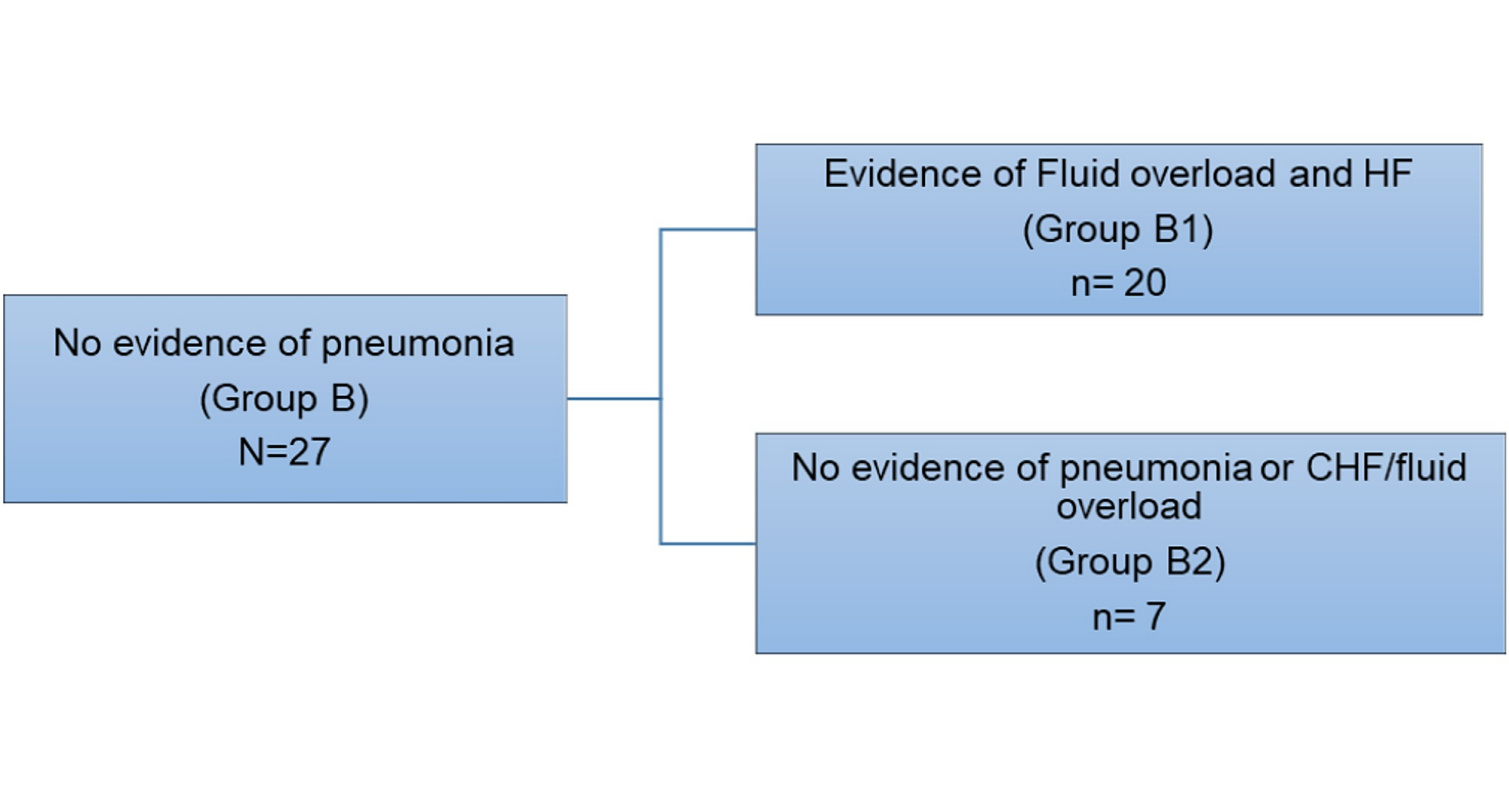
Patients in Group B (no evidence of pneumonia), further divided into two subgroups termed heart failure/fluid overload (Group B1), and no evidence of HF, pneumonia or fluid overload (Group B2) HF: heart failure; CHF: congestive heart failure

Upon analysis of echocardiogram results during hospitalization, patients in Group B (no evidence of pneumonia) were further classified into two subgroups. These were: Group B1 - heart failure/fluid overload, and Group B2 - no evidence of HF, pneumonia, or fluid overload (Figure 2). Patients with evidence of pneumonia (Group A) were compared with the group with fluid overload and heart failure (Group B1). The median BNP for both groups was 514 (IQR: 1077) vs. 1040 (2094) pg/mL, p=0.04. The median duration of inpatient antimicrobial therapy was 7 (IQR: 6) vs. 6 (IQR:4) days, p=0.60, while the median duration of the total (inpatient and discharge prescriptions) antimicrobial therapy was 11 (IQR: 6) vs. 11 (IQR: 6), p=0.32. The median duration of hospital length of stay was 9 (IQR: 9) vs. 6 (IQR:5) days, p=0.24. There were four compared to 14 patients in Groups A and B1, respectively, with documented echocardiographic evidence of ejection fraction < 55% (Table 4). Additionally, patients with pneumonia (Group A) were compared to the group with no evidence of fluid overload or pneumonia or heart failure (Group B2). The median BNP was 514 (IQR: 1077) vs. 189 (418) pg/mL p=0.02, for Groups A and B2, respectively. The median duration of inpatient antimicrobial therapy was 7 (IQR: 6) vs. 6 (IQR:4) days, p=0.61. In contrast, the median duration of the total (inpatient and discharge prescriptions) antimicrobial therapy was 11 (IQR: 6) vs. 12 (IQR: 7), p=0.24, for Groups A and B2, respectively. The median duration of hospital length of stay was 9 (IQR: 9) vs. 5 (IQR:8) days for Groups A and B2, respectively, p=0.27 (Table 5).

**Table 4:** Subgroup analysis of patients with evidence of pneumonia compared to the group with fluid overload and heart failure. BNP: brain natriuretic peptide; LVEF: left ventricular ejection fraction

Data assessed	Evidence of pneumonia (Group A)	Evidence of fluid overload and heart failure (Group B1)	P-value
Patients, N (%)	29 (51)	20 (36)	N/A
BNP (pg/mL)	514 (141-1218)	1040 (406-2500)	0.04
Duration of inpatient antimicrobial therapy (days)	7 (4-10)	6 (5-9)	0.60
Total duration of antimicrobial therapy (days)	11 (8-14)	10 (6-12)	0.24
Hospital length of stay (days)	9 (4-13)	6 (5-10)	0.32
Positive Clostridium difficile toxin	-	1 (1.78)	N/A
LVEF < 55%, N (%)	4	14	N/A

**Table 5:** Subgroup analysis comparing patients with evidence of pneumonia to the group with no evidence of fluid overload or pneumonia or heart failure BNP: brain natriuretic peptide

Data assessed	Evidence of pneumonia (Group A)	No evidence of fluid overload, pneumonia or heart failure (Group B2)	P-value
Patients, N (%)	29 (51)	7 (13)	N/A
BNP (pg/mL)	514 (141-1218)	189 (38-456)	0.02
Duration of inpatient antimicrobial therapy (days)	7 (4-10)	6 (4-8)	0.61
Total duration of antimicrobial therapy (days)	11 (8-14)	12 (11-18)	0.24
Hospital length of stay (days)	9 (4-13)	5 (3-11)	0.27

## Discussion

The study demonstrated a 48% inappropriate use of antimicrobial therapy in non-pneumonia cases with fluid congestion misdiagnosed and treated as pneumonia. These results were based on a combined analysis of clinical presentations, vital signs, radiographic evidence, microbiology, and echocardiographic data. Chest X-rays analyzed for each patient were obtained on the first day of admission, during the entire hospitalization. The rationale for this was to reduce confounding factors such as dehydration. Dehydration may be associated with the absence of infiltrates on chest X-rays, particularly on the first day of admission [16].

The study detected a difference between the BNP levels of patients with pneumonia and those with fluid overload and heart failure. This demonstrates that BNP levels, vital signs, and radiographic evidence should be used to reduce the inappropriate use of antimicrobial therapy in patients with fluid congestion. Additionally, there was no statistically significant difference in the procalcitonin levels when the group with evidence for pneumonia was compared to the group with no evidence of pneumonia. However, procalcitonin levels in the group with pneumonia was higher than the other group. Procalcitonin is a marker used to modulate antimicrobial therapy. Appropriate monitoring of clinical status, microbiology, and laboratory data are essential to ensure proper de-escalation of antimicrobial therapy.

The study had some limitations. First, data on clinical status was based on documentation in the EMR, which might not contain all the information needed to assess the patient's' status in real-time. This made it difficult to comprehend the physicians' motives for prolonged use of antimicrobial therapy, irrespective of microbiology results. Additionally, the radiographic evidence used was based only on the radiologist's impression of the chest X-rays. Moreover, BNP has been utilized as one of the markers for the CHF, but it has been shown to be elevated in other conditions. However, if the study was conducted prospectively, there would have been a better understanding of the physicians' interpretation of the chest X-ray result. Finally, there were few cases of pharmacist-led medication reconciliation; nurses reconciled most. While not the study's primary aim, the proposed intervention demonstrates avenues to expand the clinical pharmacists' roles in the ICU, emergency department, and internal medicine.

The emergency department pharmacist could perform complete medication reconciliation by interviewing patients, confirming the medication lists from the pharmacies and outpatient clinics. Clinical pharmacists in the medicine units could perform these medication reconciliations upon admission and during discharge to ensure patients are on appropriate medications for the transitions of care. The ICU pharmacists and clinical pharmacists in the medical wards could perform a daily review of antimicrobial therapy to ensure timely de-escalation or appropriate treatment for other conditions. Additionally, procalcitonin levels could be employed to modulate therapies and achieve positive outcomes. In a teaching institution such as ours and others, medical residents and students can collaborate with the antimicrobial stewardship programs to eliminate inappropriate therapies. 

## Conclusions

This study highlights the need for deployment and appropriate interpretation of radiographic evidence and several other relevant clinical parameters in the management of pneumonia in the critical care setting. The use of antimicrobial therapies through employment of radiographic evidence and clinical parameters following clinical guidelines for managing pneumonia is vital. Employment of proper antimicrobial stewardship eliminates pitfalls associated with inappropriate use of antimicrobial therapy in the setting of fluid congestion and heart failure. This will improve outcomes, reduce unnecessary drug therapies, avoid costs, drug complications, and length of hospital stay.
